# Investigating *Agrobacterium*-Mediated Transformation of *Verticillium albo-atrum* on Plant Surfaces

**DOI:** 10.1371/journal.pone.0013684

**Published:** 2010-10-27

**Authors:** Claire J. Knight, Andy M. Bailey, Gary D. Foster

**Affiliations:** School of Biological Sciences, University of Bristol, Bristol, United Kingdom; University of California Riverside, United States of America

## Abstract

**Background:**

*Agrobacterium tumefaciens* has long been known to transform plant tissue in nature as part of its infection process. This natural mechanism has been utilised over the last few decades in laboratories world wide to genetically manipulate many species of plants. More recently this technology has been successfully applied to non-plant organisms in the laboratory, including fungi, where the plant wound hormone acetosyringone, an inducer of transformation, is supplied exogenously. In the natural environment it is possible that *Agrobacterium* and fungi may encounter each other at plant wound sites, where acetosyringone would be present, raising the possibility of natural gene transfer from bacterium to fungus.

**Methodology/Principal Findings:**

We investigate this hypothesis through the development of experiments designed to replicate such a situation at a plant wound site. *A. tumefaciens* harbouring the plasmid pCAMDsRed was co-cultivated with the common plant pathogenic fungus *Verticillium albo-atrum* on a range of wounded plant tissues. Fungal transformants were obtained from co-cultivation on a range of plant tissue types, demonstrating that plant tissue provides sufficient *vir* gene inducers to allow *A. tumefaciens* to transform fungi *in planta*.

**Conclusions/Significance:**

This work raises interesting questions about whether *A. tumefaciens* may be able to transform organisms other than plants in nature, or indeed should be considered during GM risk assessments, with further investigations required to determine whether this phenomenon has already occurred in nature.

## Introduction

*Agrobacterium tumefaciens* is a specialized plant pathogen that has evolved a complex mechanism for transforming many hundreds of plant species in order to provide a unique chemical and environmental habitat for itself. The transfer of DNA from *Agrobacterium* species to plants (which in the case of *A. tumefaciens* results in production of crown galls) represents the only accepted example of common natural trans-kingdom gene transfer. Virulent *A. tumefaciens* strains possess an extrachromosomal tumour inducing (Ti) plasmid, typically ∼200 kb in size. This plasmid contains a number of features important to the transformation process, one of which is the region to be transferred, called T-DNA (transferred DNA). Through the detection of phenolic compounds produced by a wounded plant, the bacterium's virulence system is activated and T-DNA is transferred to the nuclear genome of the plant. T-DNA is delimited by a left and right border each consisting of 25 bp repeat sequences, and it is the presence of these borders, not the sequence between them that is crucial for transfer [1]. This remarkable feature has led to the development of *A. tumefaciens* as a vital tool for genetic engineering [2], [3]. In theory any piece of DNA can be placed between the borders and will be transferred, upon infection, to the plant. However, it should be noted that in nature the Ti plasmids of *A. tumefaciens*, while diverse in nucleotide sequence, all contain a relatively-conserved set of genes that code for a specific set of amino acids required by the pathogen.

Bundock *et al*., [4] demonstrated that the repertoire of organisms amenable to ATMT could be extended if the phenolic wound hormone acetosyringone was added *in vitro* to induce the virulence pathway. They reported successful *A. tumefaciens*-mediated transformation (ATMT) in the laboratory of the yeast *Saccharomyces cerevisiae* [4], with transformation only possible in the presence of acetosyringone. Experiments investigating the effect of various *vir* mutants confirmed that the mechanism for transfer uses the same *vir* machinery as for plant transformation [5], [6]. Following the publication of de Groot *et al*., [5], an ever increasing number of fungal species are proving amenable to ATMT in the laboratory [7], [8], [9], [10], [11], [12], [13], [14]. This discovery that non-plant species could also be amenable to ATMT in the laboratory demonstrates that the barriers to HGT via *A. tumefaciens* are not as strict as was first thought. Indeed it has even been reported that *A. tumefaciens* is able to transform human cells in the laboratory [15]. The *A. tumefaciens* virulence system has evolved to respond to phenolic wound hormones such as acetosyringone, and is able to transform a range of fungal species if acetosyringone is provided *in vitro*. As *A. tumefaciens* is a soil-dwelling pathogen that often infects plants through wound sites, it is conceivable that it could encounter numerous species of microorganisms at such an environmental niche, including plant pathogenic fungi also utilizing this method of plant entry. Such wound sites are likely to be exuding wound hormones such as acetosyringone, so the bacteria are likely to be primed for T-DNA transfer. This paper investigates the possibility that ATMT of fungi could be happening at such wound sites in nature. The wilt-causing fungus *Verticillium albo-atrum* is a strong candidate for encountering Agrobacterium *in planta*, as it has a similar wide host range, infecting root and crown. It has a well characterised disease cycle, is amenable to culture in the laboratory, and has recently been transformed via ATMT [16]. Perhaps most importantly, *V. albo-atrum* cannot be transformed by Agrobacterium in the absence of acetosyringone [16], therefore if Agrobacterium and *V. albo-atrum* are present together on plant tissue and transformation does occur, the acetosyringone will have been plant derived.

## Materials and Methods

### Fungal and bacterial strains

*A. tumefaciens* strain LBA1126 containing the binary vector pCAMDsRed [12] was used in all experiments. pCAMDsRed contains the DsRed (Express) gene under the control of the *Aspergillus nidulans gpd*A promoter and the *trp*C terminator, and the hygromycin B resistance gene (*hph*) under the control of a *trp*C promoter. *A. tumefaciens* cultures were prepared as in Knight *et al*., [16], with the modifcation that following 48 hour incubation in LB, cultures were diluted with sterile water to an OD_600 nm_ of 0.25, instead of being induced with acetosyringone in Induction Medium.

Conidia from wild type *V. albo-atrum* strain 1974, (Warwick-HRI) were harvested from potato dextrose agar (PDA) plates and diluted to a concentration of 10^8^ spores.ml^−1^ in sterile water.

### Preparation of plant tissues

Potato tubers (Maris Piper) and carrots (Scarlet Nantes) were aseptically peeled, sliced (approximately 30 mm diameter and 5 mm thickness) and surface sterilised in a 10% sodium hypochlorite solution. Tissue was then either placed on plates containing Murashige and Skoog (MS) agar [17] +1.5% sucrose with or without 200 µM acetosyringone, or was placed in petri dishes in the absence of any media.

Tobacco plantlets (*Nicotiana tabacum* var. White Burley) were grown in sterile magenta pots containing MS agar supplemented with 1.5% sucrose. Sections of leaf (average area 120 mm^2^) were removed and inoculated with bacterial/fungal suspension before 3–5 sections were placed on each MS+1.5% sucrose plate.

Tobacco plants (*N. tabacum* var. White Burley) approximately 8 weeks old had all leaves removed and the stem cut into sections approximately 5 cm long. These were then surface sterilised as described above. One section of stem was placed in each petri dish, in the absence of any media and a sterile scalpel used to score the surface.

### Inoculation of plant material with *A. tumefaciens* and *V. albo-atrum*

*V. albo-atrum* conidial suspension (*ca.* 10^8^ spores.ml^−1^) was mixed with an equal volume of *A. tumefaciens* culture (an OD_600 nm_ of 0.25–0.30) before being inoculated onto plant tissue. For potato slice experiments, either 40 µl or 200 µl was pipetted onto the surface, for carrot slices the volume was 40 µl. For tobacco stem experiments, 100 µl suspension was inoculated onto the surface of stems. Leaf experiments involved placing leaf sections in a 1∶1 suspension of conidia and *A. tumefaciens* for 5 minutes. Control plates for each experiment comprised plant material inoculated solely with wild type *V. albo-atrum*.

Plates were left at room temperature for a minimum of 8 days and a maximum of 42 days.

### Harvesting of samples

A range of methods were attempted with the early experiments (potato and carrot) in order to successfully isolate putative transformants from plant tissue. These included using an inoculating loop to disrupt the previously inoculated surface of the plant tissue in the presence of ∼2 ml sterile water. This liquid was then pippetted onto selection plates (PDA+50 µg.ml^−1^ hygromycin B and 75 µg.ml^−1^ Timentin). Alternatively, plant material was removed from the co-cultivation plate and homogenised in an autoclaved pestle and mortar, before either being added to molten selection media, or spread over selection media plates. In the case of experiments with tobacco stems, stem sections were cut in half longitudinally before being placed (cut side down) onto selection plates.

For potato slices, transformants were obtained using one of three methods: by homogenising the plant material in a pestle and mortar and either spreading the suspension directly on selection plates (PDA+50 µg.ml^−1^ hygromycin B and 75 µg.ml^−1^ Timentin) or by adding the suspension to molten selection media and pouring into a plate, or by disrupting the plant tissue using a sterile inoculating loop and ∼2 ml sterile water and adding the suspension to molten selection media before pouring into a plate.

For tobacco leaf experiments, plant tissue was homogenised in pestle and mortars before being spread over selection plates. In the case of experiments with tobacco stems, stem sections were cut in half longitudinally before being placed (cut side down) onto selection plates.

### Microscopy

Putative transformants were screened for DsRed expression *in situ* and following subculture onto fresh selection plates using a DM-LB Leica microscope fitted with a red-shifted TRITC filter (excitation 545/30 nm, emission 620/60 nm) [18], [19].

### DNA extraction and analysis

Following repeated subculturing onto selection plates to obtain single colonies, fungal genomic DNA was extracted essentially as described in Keon and Hargreaves [20] (see [21]). PCR amplification was then conducted on genomic DNA using primers HygF (5′-GCGTGGATATGTCCTGCGGG-3′) and HygR (5′-CCATACAAGCCAACCACGG-3′) to produce a 598 bp fragment of the *hph* gene [22]. For confirmation of presence of the DsRed gene, primers DsRedF (5′-AGGACGTCATCAAGGAGTTC-3′) and DsRedR (5′-CAGCCCATAGTCTTCTTCTG-3′) resulted in the amplification of a 414 bp fragment. Species specific ITS primers - VaaF (5′-CCGCCGGTACATCAGTCTCTTTATTTATAC-3′) and VaaR (5′- GTGCTGCGGGACTCCGATGCGAGCTGTAAT-3′) that amplify a ∼300 bp ITS region were used to confirm fungal DNA was of *V. albo-atrum* origin [23].

To determine T-DNA copy number, Southern analysis was performed by digesting 10 µg genomic DNA with *Xba*I, before being separated via electrophoresis and transferred to Hybond N^+^ membrane. Standard protocols [24] were used for hybridisation using a [α-^32^P] dCTP labeled DsRed PCR product.

## Results

### Transformation of *V. albo-atrum* by *A. tumefaciens* on carrot and potato tissues

**Figure 1:**
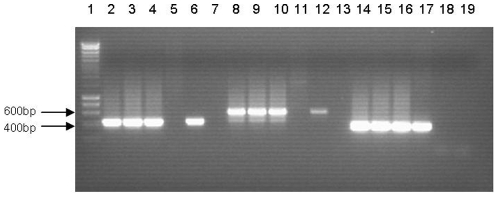
PCR analysis of 3 *V. albo-atrum* transformants obtained from ATMT experiments on potato. Lane 1, DNA Hyperladder I (Bioline), lanes 2–7, PCR with DsRed primers; 3 transformants (2–4), wild type *V. albo-atrum* (5), pCAMDsRed (6), water (7). Lanes 8–13, PCR with hygromycin B primers; 3 transformants (8–10), wild type *V. albo-atrum* (11), pCAMDsRed (12), water (13). Lanes 14–19, PCR with wild type *V. albo-atrum* ITS primers; 3 transformants (14–16), wild type *V. albo-atrum* (17), pCAMDsRed (18), water (19).

**Figure 2:**
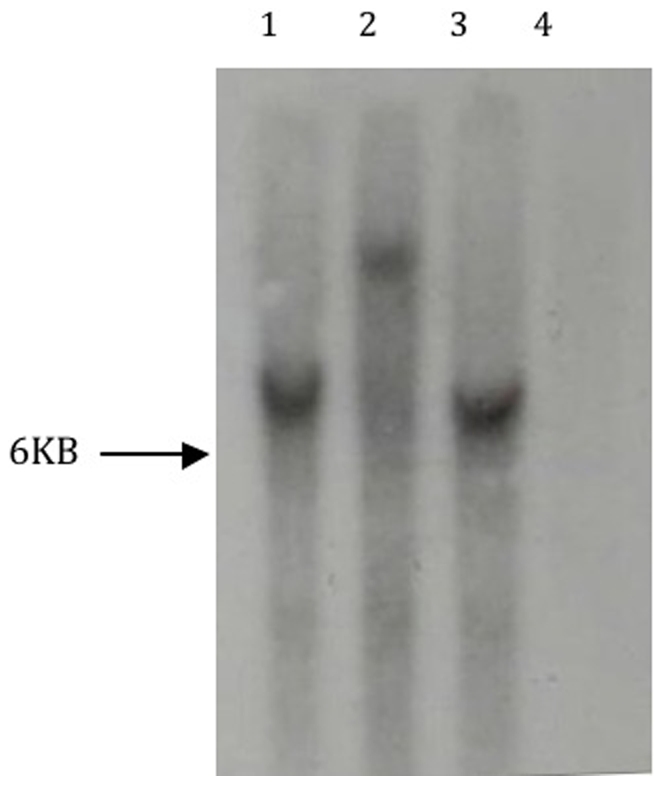
Autoradiograph showing Southern analysis of 3 transformants obtained from potato slice hosts. 10 µg genomic DNA was digested with *Xba*I and probed with the 414 bp fragment of the DsRed gene obtained via PCR of pCAMDsRed using primers DsRed forward and reverse. Lanes 1–3, transformants, lane 4, wild type *V. albo-atrum*.

Potato tubers and carrots are both natural hosts of *V. albo-atrum* [25] and are easy to obtain and sterilise. The first transformants were obtained from control experiments, with co-cultivation with *A. tumefaciens* and *V. albo-atrum* on a potato slice, where acetosyringone was present in the MS co-cultivation medium. This demonstrated that the selection conditions worked, and that the plant tissue in this system was not in any way inhibitory to the ATMT of fungi. Following this initial success, ‘more natural’ experiments were conducted, whereby no exogenous acetosyringone was provided other than that produced by the wounded plant tissue. From these conditions, 3 transformants, 2 from potato slices (17 plates, 1 slice per plate), and 1 from carrot (15 plates, 1 slice per plate) were isolated. All putative transformants were subcultured onto fresh selection plates to confirm hygromycin B resistance, and screened using microscopy for DsRed expression. Transformants maintained hygromycin B resistance and DsRed expression following repeated subculturing in both the presence and absence of selection suggesting that stable transformation had occurred. No hygromycin B resistant, DsRed expressing colonies were obtained in any of the controls, where just fungal material was inoculated onto plant tissue in the absence of *A. tumefaciens*. PCR was performed on the transformants generated on potato disc, which confirmed presence of both the *hph* and DsRed genes in all transformants (see Figure 1), and Southern analysis demonstrated that as with conventional ATMT of *V. albo-atrum* [16] T-DNA integrated randomly into genomic DNA, and as single copy insertions (Figure 2).

### Transformation of *V. albo-atrum* by *A. tumefaciens* on tobacco stem and leaf tissues

Following successful isolation of fungal transformants from potato and carrot slices, tobacco leaves were investigated as another example of a semi-natural damaged area upon which ATMT of a fungus may occur in the absence of exogenously provided acetosyringone. A total of 14 transformants were obtained from four replicated experiments (comprising 42 plates, 3–5 leaf pieces per plate). In all four experiments the only source of acetosyringone was the plant tissue and *A. tumefaciens* had not been pre-induced. Control plates containing just plant tissue inoculated with *V. albo-atrum* never resulted in hygromycin B resistant DsRed expressing transformants. Putative fungal transformants were screened via DsRed microscopy and repeated subculturing on selection plates, before a subset of 10 were shown to be transformed by PCR for both DsRed and hygromycin and Southern analysis (data not shown).

Finally, following successful ATMT of *V. albo-atrum* on tobacco leaves in the absence of exogenous acetosyringone, further experiments even more representative of a ‘natural’ situation for ATMT of *V. albo-atrum* were conducted. *A. tumefaciens* was co-cultivated with *V. albo-atrum* on and within tobacco stems in the absence of any medium on the plates. The lack of agar medium on the plate means that the fungus and bacteria are confined within/on the dying stem section for survival, and any acetosyringone produced could not diffuse into the medium.

**Figure 3:**
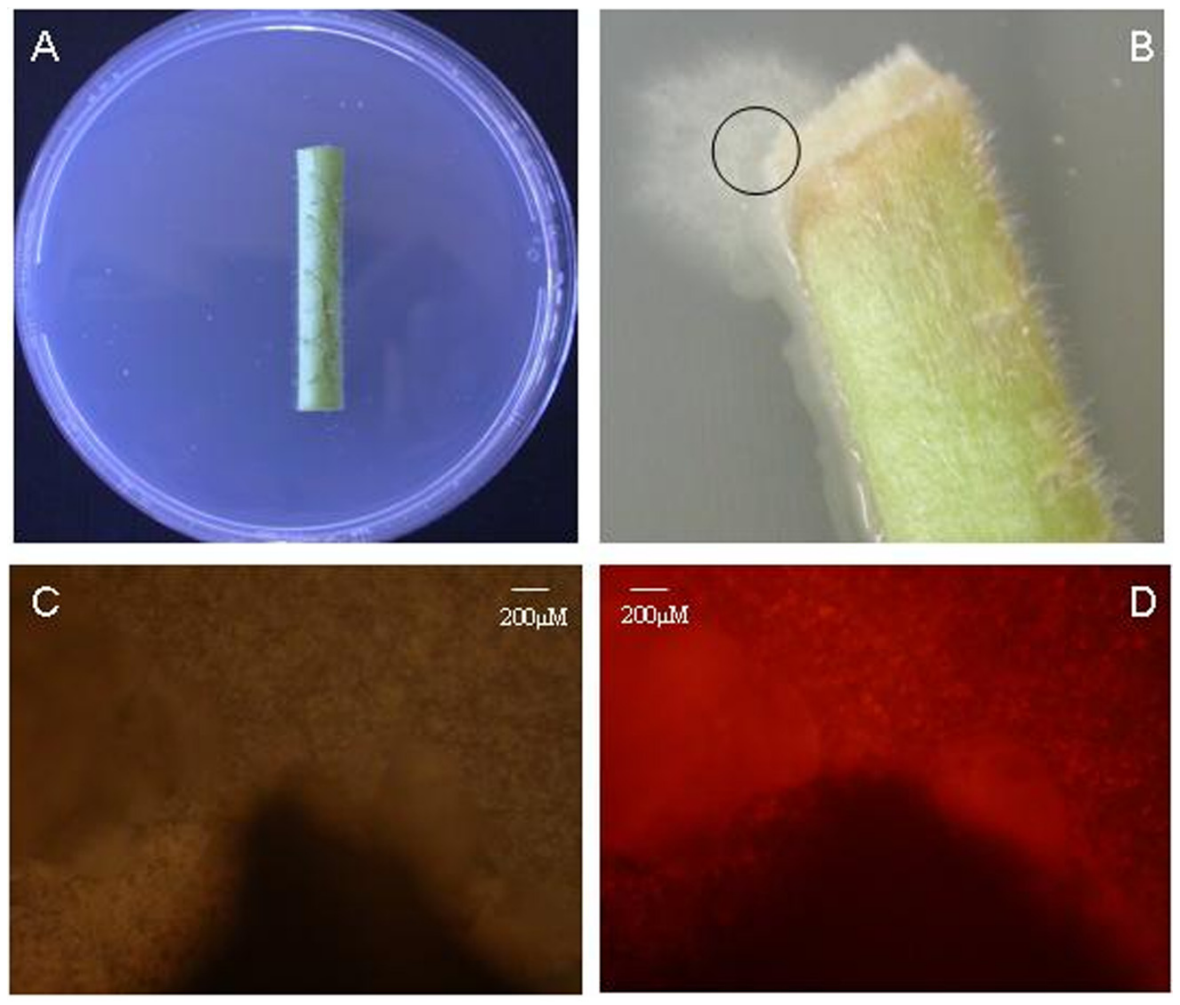
ATMT of *V. albo-atrum* on stems. Stem section prior to inoculation (Panel A), (Panel B) stem section showing a transformed *V. albo-atrum* colony emerging from the stem onto the selection medium ten days after transfer to selection (stems were co-cultivated on no media for 12–22 days prior to selection), (Panel C) white light microscopy of plant/PDA interface circled in B, (Panel D) the same region viewed under the DsRed TRITC filter.

Co-cultivation of *A. tumefaciens* with *V. albo-atrum* conidia on tobacco stems (Fig. 3A) resulted in the isolation of 10 fungal transformants in total from two independent experiments (31 plates in total, 1 stem per plate). Transformants were visualised *in situ* (Figure 3B,C and D) with all demonstrating strong fluorescence before being subcultured to fresh selection plates, and again confirmed positive via PCR analysis (data not shown). No transformants were observed on control plates.

## Discussion

It is well established that the plant pathogenic bacterium *A. tumefaciens* transforms plants in nature, producing crown galls as a result of the transfer of T-DNA to the plant genome [26]. This natural form of genetic modification has been adapted in laboratories over the last 20 years to enable the insertion of almost any foreign gene into plants [27]. More recently it has been found that non-plant organisms such as fungi, and even human cells can be transformed via ATMT in the laboratory. We investigated whether ATMT of non-plant organisms could happen *in planta*, with induction of *A. tumefaciens* virulence system being provided by damaged plant tissue. The plant pathogenic fungus *V. albo-atrum* was chosen as an initial test system because such fungi have the potential to co-exist on plant surfaces with the possibility of colonising microenvironments such as stems and crown galls, with *V. albo-atrum* and *A. tumefaciens* commonly causing disease on the same plant species such as hops, rose and raspberry. *V. albo-atrum* has also recently been transformed via ATMT in the laboratory [16] further confirming its suitability for such studies.

In the case of fungi at least, transformation using *A. tumefaciens* appears to require the addition of acetosyringone [9], [10], [12], [16]. We recently demonstrated this to also be true in the case of *V. albo-atrum* [16] with no transformants being produced if acetosyringone was not provided exogenously. Initial experiments on plant tissue therefore included acetosyringone provided in the co-cultivation medium. The production of transformants from such conditions demonstrated that selection conditions were suitable, and that plant material was not in any way inhibitory to ATMT of fungi.

This led to the development of further experiments designed to mimic a more natural situation, with the only source of acetosyringone being the damaged plant tissue. Consequently, *V. albo-atrum* transformants were successfully obtained following co-cultivation of *A. tumefaciens* and *V. albo-atrum* on carrot and potato slices, tobacco leaves and stems. These reproducible findings appear to be the first evidence that *A. tumefaciens* is capable of transforming a fungus in a semi-natural environment, and suggests that in principle at least, this could also be possible in nature.

Whilst transformation is less efficient than in conventional ATMT of *V. albo-atrum* [16], the relative ease (following optimisation) with which fungal transformants were obtained from a range of plant material indicates that there is a possibility that this could be occurring in nature.

This study focused on ATMT of *V. albo-atrum in planta*, however there are numerous other fungi that *A. tumefaciens* could also encounter at wound sites in nature (for example *Fusarium oxysporum*) that could have been used in this study, and which may also be amenable to ‘semi-natural’ ATMT. The isolation of fungal transformants following co-cultivation on several types of plant tissue – carrot, potato and tobacco – may also indicate that not only is ATMT of *V. albo-atrum* possible on different parts of a plant, but also on a range of different host plants.

This work therefore raises interesting questions about whether the host range of *A. tumefaciens* in nature is greater than just plants. It is possible that evidence of such events could be looked for retrospectively in the increasing number of genome sequences becoming available. Machida *et al*., [28] reported the finding of two genes of possible *Agrobacterium* origin in the genome sequence of *Aspergillus oryzae*, although as they resemble chromosomal genes rather than T-DNA the mechanism for such transfer is unclear. Nevertheless, there have been reports of similar transfers of *Agrobacterium* chromosomal DNA being transferred to plants in the absence of any obvious T-DNA signatures [29]. Therefore such hypothetical transfer events warrant further investigation.

If this is indeed a possible natural form of horizontal gene flow previously unappreciated it is interesting purely from an evolutionary point of view. However, the biological significance of any such gene transfers may depend on whether the transferred DNA confers any selective advantage to the organism in its natural environment.

In addition, the results may well have implications for the risk assessment of GM plants generated via Agrobacterium-mediated transformation, as Agrobacterium can survive within plant tissue through transformation and tissue culture and can therefore be found within regenerated transgenic plants [30], [31]. Much has been made of the fact that the elimination of Agrobacterium is essential to prevent this as a possible route of gene escape possibly from Agrobacterium to other bacteria, but this study shows that the encounter between Agrobacterium and a plant pathogenic fungus on a plant surface can lead to gene flow in a new, and to-date, under investigated way.

Indeed, many susceptible crop plants are thought to be infected by Agrobacterium primarily during propagation, especially perennial crops that are grafted (e.g., rose), and it is clear that more and more GM perennial crops are being generated and grown each year.

In conclusion, we have shown that a range of plant tissues are capable of inducing *A. tumefaciens* to transfer T-DNA to the plant pathogenic fungus *V. albo-atrum* in a range of semi-natural experiments reminiscent of plant wound/damage sites. This suggests that *A. tumefaciens* may be able to transform non-plant organisms such as fungi in nature, the implications of which are unknown.
